# Change of prescription for patients with schizophrenia or major depressive disorder during admission: real-world prescribing surveys from the effectiveness of guidelines for dissemination and education psychiatric treatment project

**DOI:** 10.1186/s12888-023-04908-4

**Published:** 2023-06-28

**Authors:** Naoki Hashimoto, Norio Yasui-Furukori, Naomi Hasegawa, Shuhei Ishikawa, Hikaru Hori, Hitoshi Iida, Kayo Ichihashi, Kenichiro Miura, Junya Matsumoto, Shusuke Numata, Fumitoshi Kodaka, Ryuji Furihata, Kazutaka Ohi, Kazuyoshi Ogasawara, Jun-ichi Iga, Hiroyuki Muraoka, Hiroshi Komatsu, Masahiro Takeshima, Kiyokazu Atake, Mikio Kido, Toshinori Nakamura, Taishiro Kishimoto, Akitoyo Hishimoto, Toshiaki Onitsuka, Tsuyoshi Okada, Shinichiro Ochi, Tatsuya Nagasawa, Manabu Makinodan, Hiroki Yamada, Takashi Tsuboi, Hisashi Yamada, Ken Inada, Koichiro Watanabe, Ryota Hashimoto

**Affiliations:** 1 Department of Psychiatry, Hokkaido University Graduate School of Medicine, Hokkaido, Japan; 2 Department of Psychiatry, Dokkyo Medical University, School of Medicine, Mibu, 321-0293 Shimotsuga, Tochigi Japan; 3 Department of Pathology of Mental Diseases, National Institute of Mental Health, National Center of Neurology and Psychiatry, Tokyo, Japan; 4 Department of Psychiatry, Faculty of Medicine, Fukuoka University, Fukuoka, Japan; 5 Department of Neuropsychiatry, University of Tokyo Hospital, Tokyo, Japan; 6 Department of Psychiatry, Graduate School of Biomedical Science, Tokushima University, Tokushima, Japan; 7 Department of Psychiatry, The Jikei University School of Medicine, Tokyo, Japan; 8 Agency for Student Support and Disability Resources, Kyoto University, Kyoto, Japan; 9 Department of Psychiatry, Gifu University Graduate School of Medicine, Gifu, Japan; 10 Center for Postgraduate Clinical Training and Career Development, Nagoya University Hospital, Nagoya, Japan; 11 Department of Neuropsychiatry, Molecules and Function, Ehime University Graduate School of Medicine, Ehime, Japan; 12 Department of Psychiatry, Kitasato University School of Medicine, Sagamihara, Japan; 13 Department of Psychiatry, Tohoku University Hospital, Sendai, Japan; 14 Department of Neuropsychiatry, Akita University Graduate School of Medicine, Akita, Japan; 15 Health Administration Center (Kyusyu region), Nippon Telegraph and Telephone West Corporation, Fukuoka, Japan; 16 Kido Clinic, Toyama, Japan; 17 Department of Psychiatry, Shinshu University School of Medicine, Matsumoto, Japan; 18 Hills Joint Research Laboratory for Future Preventive Medicine and Wellness, Keio University School of Medicine, Tokyo, Japan; 19 Department of Psychiatry, Kobe University Graduate School of Medicine, Kobe, Japan; 20 National Hospital Organization Sakakibara Hospital, Tsu, Japan; 21 Department of Psychiatry, Jichi Medical University, Shimotsuke, Japan; 22 Department of NeuroPsychiatry, Kanazawa Medical University, Ishikawa, Japan; 23 Department of Psychiatry, Nara Medical University, Nara, Japan; 24 Department of Psychiatry, Showa University Northern Yokohama Hospital, Yokohama, Japan; 25 Department of Neuropsychiatry, Kyorin University School of Medicine, Tokyo, Japan; 26 Department of Neuropsychiatry, Hyogo Medical University, Hyogo, Japan

**Keywords:** Antipsychotics, Antidepressants, Monotherapy, Polypharmacy, Psychotropics

## Abstract

**Background:**

Polypharmacy of additional psychotropics alongside the main treatment drug (antipsychotics in schizophrenia and antidepressants in major depressive disorder) is common in Japan. Our goal is to align psychotropic prescription in Japan with international standards, while reducing the differences between facilities. To achieve this goal, we aimed to compare prescriptions at the time of hospital admission and discharge.

**Methods:**

Data on prescriptions at admission and discharge from 2016 to 2020 were collected. We divided the patients into four groups: (1) mono_mono group, monotherapy of the main drug at admission and discharge; (2) mono_poly group, monotherapy at admission and polypharmacy at discharge; (3) poly_poly group, polypharmacy at admission and discharge; and (4) poly_mono group, polypharmacy at admission and monotherapy at discharge. We compared the changes in dosage and number of psychotropics among the four groups.

**Results:**

For both schizophrenia and major depressive disorder, the patients who received monotherapy with the main drug at admission were likely to receive main drug monotherapy at discharge and vice versa. For schizophrenia, the polypharmacy was prescribed more often in the mono_poly group than that in the mono_mono group. The prescription was not changed at all for more than 10% of the patients.

**Conclusions:**

It is critical to avoid a polypharmacy regimen to ensure that guideline-compliant treatment is provided. We expect higher rates of monotherapy with the main drug after the EGUIDE lectures.

**Trial registration:**

The study protocol was registered in the University Hospital Medical Information Network Registry (UMIN000022645).

**Supplementary Information:**

The online version contains supplementary material available at 10.1186/s12888-023-04908-4.

## Introduction

Second-generation antipsychotic monotherapy with no or limited concurrent use of other psychotropics is recommended in the pharmacotherapy of patients with schizophrenia in most guidelines [1, 2] including that in the recently revised Japanese treatment guidelines for schizophrenia [3]. However, prescribing two or more antipsychotics concurrently (antipsychotic polypharmacy) and concurrent use of psychotropic agents in the treatment of schizophrenia are common clinical practices [4, 5]. Our recent study showed that 43.2% of the patients with schizophrenia received polypharmacy with antipsychotics [6]. This figure reflects the average for the 15 Asian countries that joined the Fourth Research on Asian Psychotropic Prescription Pattern (REAP-4AP) project [5] and is higher than the polypharmacy rate reported for Western countries [7]. In the same study, 66.5% of the patients were concurrently administered anxiolytics and hypnotics, which was much higher than that observed in the REAP-4AP data (average: 27.9% for anxiolytics and 9.3% for hypnotics). In addition, the concomitant use of anti-Parkinson’s drugs, anxiolytics, and hypnotics was more often associated with antipsychotic polypharmacy than monotherapy [6].

For major depressive disorder (MDD), antidepressant monotherapy is the first-line treatment in most clinical guidelines [8, 9]. In our study, the percentages of antidepressant polypharmacy, concomitant anxiolytic and hypnotic use, and concomitant second generation antipsychotic use by patients with MDD were 25.4%, 74.4%, and 54.5%, respectively [6]. These findings highlight that in line with recent studies from western countries [10, 11], most patients receive antidepressant polypharmacy or concomitant use of other psychotropics. Notably, anxiolytics and hypnotics are not evidence-based augmentation options [9] The large difference in prescriptions among institutions is another challenge, as the rate of antidepressant monotherapy ranged from less than 20–100% [12].

In Japan, the Effectiveness of Guidelines for Dissemination and Education (EGUIDE) psychiatric treatment project was launched in 2016. In the EGUIDE project, we developed a 2-day education course for psychiatrists to learn Japanese treatment guidelines for schizophrenia and MDD (one day for each disorder). The EGUIDE program was shown to improve clinical knowledge [13] and practice [14] and was highly appreciated by the participants [15]. In addition, we evaluated the participants’ prescribing activity (prescription at discharge for patients with schizophrenia or MDD) to assess the effectiveness of our program. Our data showed how real-world prescriptions conform to or differ from the guidelines [16–21]. Moreover, our data indicated that antidepressant or antipsychotic polypharmacy and the concomitant use of psychotropics is still prevalent in Japan. Our next goal is to bring these figures up to the international level while reducing the differences between institutions.

To achieve our goal, we compared prescriptions at the time of hospital admission and discharge in the current study. By assessing changes in prescriptions during hospitalization, we aimed to identify the clues that improve pharmacological prescriptions for patients with schizophrenia and MDD.

## Methods

### Informed consent/ethics review

Psychiatrists were recruited between April 2016 and September 2020. Written informed consent was obtained from all the participants after the procedures had been fully explained by a chief researcher at the facility [13]. This study was approved by the ethics committees of the National Center of Neurology and Psychiatry (A2017-105) and each participating university, hospital, or clinic. The study was conducted in accordance with the Declaration of Helsinki. The study protocol was registered in the University Hospital Medical Information Network Registry (UMIN000022645).

### Prescription data

From April to September of each year between 2016 and 2020, prescriptions at admission and discharge at each participating institution were collected using a standardized data collection method. In the EGUIDE, we have collected prescription data from April to September. We have held the 2 day guideline lecture for several times throughout Japan from October to December, and have checked quality of our data from October to March. We checked the types and dosages of all psychotropics, including antipsychotics, antidepressants, anti-Parkinson drugs, hypnotics, anxiolytics, mood stabilizers, antiepileptics, and other types of drugs, such as psychostimulants or antidementia agents. Sulpiride was defined as antipsychotics, and clonazepam was defined as hypnotics, and anxiolytics. Vegetamin A (chlorpromazine 25 mg, promethazine 12.5 mg, and phenobarbital 40 mg) and Vegetamin B (chlorpromazine 12.5 mg, promethazine 12.5 mg, and phenobarbital 30 mg) were defined as hypnotics and anxiolytics. Acetylpheneturide was defined as a mood stabilizer and an anticonvulsant. Acamprosate calcium, acetazolamide, atomoxetine hydrochloride, cyanamide, diphenhydramine 40 (Travelmin®), disulfiram, donepezil, gabapentin enacarbil, galantamine, guanfacine, lisdexamfetamine mesilate, memantine, methamphetamine, methylphenidate hydrochloride (Concerta®), modafinil, nalmefene, pemoline, phenobarbital 15 (Trancolon P®), phenobarbital 20 (asthmolysin), reserpine, and rivastigmine skin patches were regarded as other types of drugs (see Supplementary Table 1).

Data were collected from October to March of each year by the EGUIDE project members. Prescription data of 2179 and 891 patients who had been diagnosed with schizophrenia and MDD, respectively, were gathered from 96 institutions (40 university hospitals, 24 national/public hospitals, and 32 private hospitals). We used the initial prescription data of each institution to evaluate prescription patterns before the 2-day intensive and comprehensive session on the guidelines. Only data that passed the quality control assessment were included in the final analysis.

Conventionally, equivalents of chlorpromazine for antipsychotics, imipramine equivalent for antidepressants, biperiden for anti-Parkinson agents, and diazepam/nitrazepam equivalent for hypnotics and anxiolytics have been used to sum and compare the daily dose of agents belonging to the same category. Drugs for with no equivalent doses were excluded from the analysis. The equivalent doses were calculated according to the study by Inada and Inagaki [22] except for several new agents [23–26] (see Supplementary Table 1).

### Statistics

All analyses were conducted using R 3.2.3 (The R Foundation for Statistical Computing, Vienna, Austria). The significance threshold was set at P < 0.05. Bonferroni correction for repeated analyses was applied to reduce type 1 errors generated by multiple comparisons, except for the post-hoc analysis. Since we performed the statistical analysis 62 times in this study, p < 0.00081 (0.05/62) was judged to be significant.

### Analysis 1 (Fig. 1A–E)

**Fig. 1:**
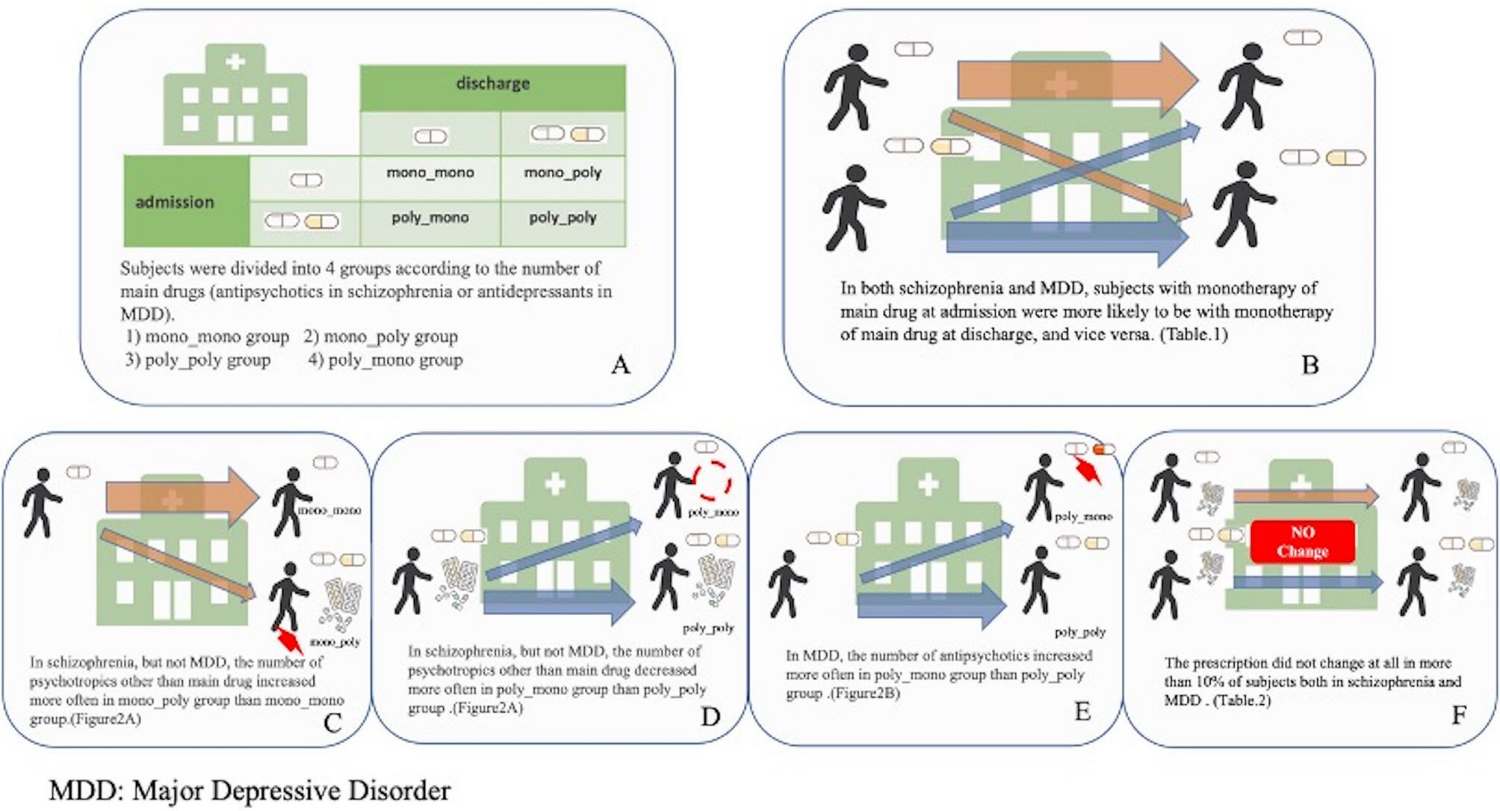
Graphical abstract of this study

We excluded patients who were not prescribed the main drug (antipsychotics for schizophrenia and antidepressants for MDD) at admission or discharge. As a result, 2098 patients with schizophrenia and 814 patients with MDD were included. We divided these patients into four groups: (1) mono_mono group, patients prescribed main drug monotherapy at admission and discharge; (2) mono_poly group, patients prescribed main drug monotherapy at admission and polypharmacy at discharge; (3) poly_poly group, patients prescribed polypharmacy of the main drugs at admission and discharge; and the (4) poly_mono group, patients prescribed polypharmacy of the main drugs at admission and monotherapy at discharge (Fig. 1A). We compared the changes in the dosage and number of psychotropics other than the main drug among the groups. Chi-squared test was used for categorical variables. Post-hoc Holm test was used after the chi-squared test if needed.

### Analysis 2 (Fig. 1F)

The next analysis included 2179 patients with schizophrenia and 891 patients with MDD. The patients were divided into two groups: (1) patients without any changes in their prescription during admission, and (2) patients with a change in their prescription during admission. Patients with a change in their prescription during admission were further divided into three groups: (2.1) change in the type of main drug, (2.2) change in the dose or number of main drugs, and (2.3) changes in the psychotropics used.

The chi-squared test was used to compare patients with schizophrenia and MDD. The post-hoc Holm test was used after the chi-squared test. Using the chi-squared test for both schizophrenia and MDD, the percentage of patients receiving modified electroconvulsive therapy (mECT) was compared between those with and without any changes in their prescription during admission. The percentage of patients in the MDD group who received cognitive behavioral therapy (CBT) was also compared.

## Results

### Analysis 1

#### Schizophrenia

The number and percentage of patients included in the four groups are shown in Table 1. The demographic background and psychotropic prescriptions of these patients at admission and discharge are shown in Supplementary Tables 2 and 3. The chi-squared test for Table 1 was statistically significant, implying that a higher ratio of patients with antipsychotic monotherapy at admission received antipsychotic monotherapy at discharge in comparison to the patients who received antipsychotic polypharmacy at admission (Table 1; Fig. 1B).

**Table 1:** Changes in the number of antipsychotics/antidepressants for subjects with schizophrenia/major depression disorder

		antipsychotics for subjects with schizophrenia^*1^		antidepressants for subjects with MDD^*1^	
		discharge		discharge	
		monotherapy	polypharmacy	monotherapy	polypharmacy
admission	monotherapy	866 (41.3%)	260 (12.4%)	508 (62.4%)	78 (9.6%)
	polypharmacy	254 (12.1%)	718 (34.2%)	92 (11.3%)	136 (16.7%)

MDD, Major depressive disorder; *1: p < 0.00001 (chi-squared test)

**Fig. 2:**
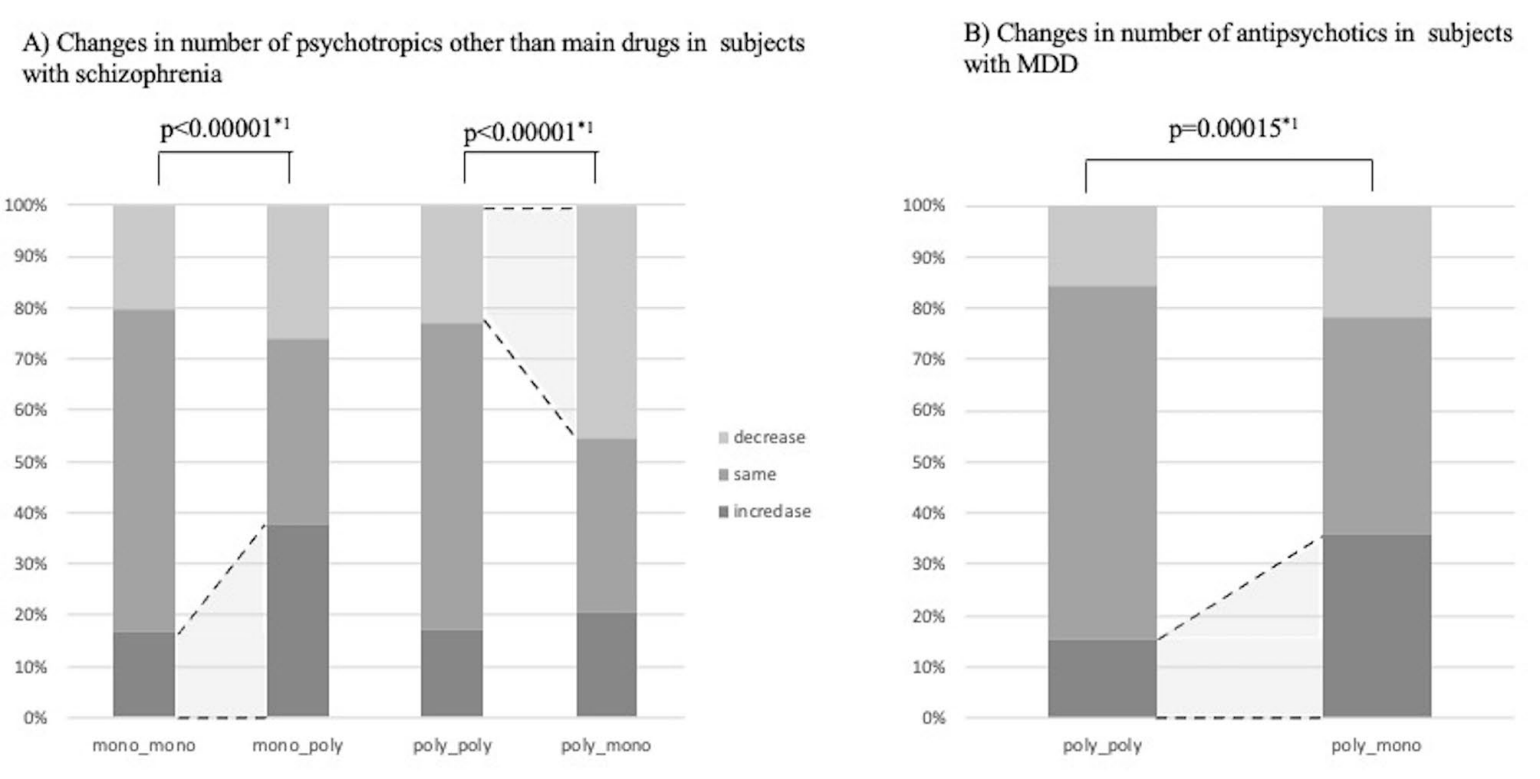
Changes in number of psychotropics other than main drugs (**A**) Result of subjects with schizophrenia: The Mono_poly group was prescribed a significantly higher number of psychotropics other than main drugs than the mono_mono group. The Poly_mono group was prescribed a significantly lower number of psychotropics other than the main drugs than the poly_poly group (**B**) Result of subjects with MDD: The Poly_mono group was prescribed more antipsychotics than the poly_poly group *1: chi-squared test MDD, major depressive disorder, mono_mono, antipsychotics/antidepressants monotherapy at admission and discharge; mono_poly, antipsychotics/antidepressant monotherapy at admission and polypharmacy at discharge; poly_mono, antipsychotics/antidepressants polypharmacy at admission and monotherapy at discharge; poly_poly, antipsychotics/antidepressants polypharmacy at admission and at discharg

The number of concurrent psychotropic drugs used increased more often in the mono_poly group than in the mono_mono group (Figs. 1C and 2A); this was also true for the number and dose of drugs in each category, except for antidepressants and other drugs (Supplementary Table 6). In contrast, the number of psychotropic drugs used decreased more often in the poly_mono group than in the poly_poly group (Figs. 1D and 2 A); this was also true for the number and dose of drugs in each category, except for antidepressants and other drugs (Supplementary Table 6).

The frequent prescribed antipsychotics for subjects with SZ in monotherapy and polypharmacy at discharge were presented in the supplementary data ( Supplementary Tables 8, 9).

#### MDD

The number and percentage of patients included in the four groups are shown in Table 2. The demographic background and prescription of psychotropics at admission and discharge are shown in Supplementary Tables 4 and 5. As in the case of schizophrenia, the chi-squared analysis results were statistically significant, meaning a higher ratio of patients with antidepressant monotherapy at admission received antipsychotic monotherapy at discharge than those with antidepressant polypharmacy at admission (Table 1; Fig. 1B).

**Table 2:** Changes in the prescription of psychotropics in subjects with schizophrenia and major depression disorder during admission

	schizophrenia (main drug: antipsychotics)	major depression disorder (main drug: antidepressants)	p value^*1^
No change in psychotropics	404 (18.5%) ^*2^	109 (12.2%) ^*2^	p < 0.00001
Any change in psychotropics	1775 (81.5%)	782 (87.8%)	
1) Change the type of main drug	860 (39.5%)	331 (37.1%)	
2) Change the dose or number of main drug without 1)	700(32.1%)^*2^	223 (25.0%)^*2^	
3) Change other psychotropics without 1), 2)	215 (9.9%)^*2^	228 (25.6%)^*2^	

n (%)

*1: chi-squared test; *2, Holm test (p < 0.05)

The change in the number of psychotropics other than the main drug did not significantly differ between the patients in the mono_mono and mono_poly groups or between patients in the poly_poly and poly_mono groups (Supplementary Table 7). Analyses of changes in the number and dose of drugs in each drug category showed that the dose of antipsychotics increased more often in the poly_mono group than in the poly_poly group. This was especially true for atypical antipsychotics, although this trend did not reach statistical significance (Figs. 1E and 2B, Supplementary Table 7).

The frequent prescribed antidepressants for subjects with MDD in monotherapy and polypharmacy at discharge were presented in the supplementary data (Supplementary Tables 10, 11).

### Analysis 2

A lack of psychotropic prescription change during admission was more common in patients with schizophrenia (18.5%) than in those with MDD (12.2%). However, a change in concurrent antipsychotics, without any change in the main drug treatment, was more common in patients with MDD (25.6%) than in those with schizophrenia (9.9%) (Fig. 1F; Table 2). In the case of schizophrenia, the percentage of patients who received mECT was higher among those whose prescription remained unchanged (12.1%) than among those whose prescriptions underwent some changes (6.3%, p = 0.00007 by chi-squared test). On the other hand, in the case of MDD, the percentage of patients who received mECT (12.8%) or CBT (0.9%) did not differ significantly between those who prescriptions remained unchanged and those whose prescriptions underwent some changes (mECT, 16.2%; CBT, 1.7%; p = 0.4412 in chi-squared test for mECT, p = 0.8612 in chi-squared test for CBT).

## Discussion

In the current study, we summarized the prescription changes during admission in 2179 patients with schizophrenia and 891 patients with MDD from 96 institutions in Japan between 2016 and 2020. To the best of our knowledge, this is the first study to examine changes in prescriptions during hospitalization in a large sample.

For both schizophrenia and MDD, patients who received monotherapy with a main treatment drug (antipsychotics for schizophrenia and antidepressants for MDD) at admission were more likely to receive monotherapy with the same drug at discharge and vice versa. As mentioned above, the Japanese guidelines for schizophrenia strongly recommend main drug monotherapy [3]. Although the effectiveness of antipsychotic poly pharmacy compared to monotherapy for schizophrenia is controversial [27], avoidance of unnecessary polypharmacy is essential to prevent and minimize potentially adverse drug reactions [28]. In the guidelines for MDD, [9] augmentation with mood stabilizers or atypical antipsychotics (but not anxiolytics and hypnotics) is recommended for patients who respond partially to their current antidepressant; notably, polypharmacy of antidepressants is allowed for patients with treatment-resistant depression. However, a recent study of 226 inpatients with depressive disorders showed that adverse drug reactions were found 2–3 times more frequently in patients treated with polypharmacy than in those treated with monotherapy [29]. Our current findings suggest that once polypharmacy is initiated as the main treatment, it is difficult to step down to monotherapy, even during hospitalization. Therefore, it is important to make every effort to avoid polypharmacy as the main treatment.

In patients with schizophrenia, the number of additional psychotropic drugs prescribed increased more often in the mono_poly group than in the mono_mono group and decreased more often in the poly_mono group than in the poly_poly group. This is in line with the findings of our previous study, which showed that concomitant use of anti-Parkinson’s drugs, anxiolytics, and hypnotics was often associated with antipsychotic polypharmacy than monotherapy [6]. However, due to the lack of strong evidence and reports of various adverse reactions, the guidelines did not recommend concomitant use of psychotropics, other than antipsychotics and short-term benzodiazepine use [2, 3]. Importantly, we should maintain patients on monotherapy as much as possible to prevent unnecessary concomitant use of psychotropics.

However, special care must be taken when interpreting the comparison of the poly_poly and poly_mono groups for schizophrenia. Our data showed that a decrease in polypharmacy to monotherapy was associated with a decrease in the number and dose of other psychotropics, and this may support aggressive reduction in the number of antipsychotics prescribed during hospitalization. On the other hand, a recent meta-analysis showed that there was a significant difference in study discontinuation due to all causes in favor of staying on antipsychotic polypharmacy rather than switching to monotherapy [30]. However, five out of six studies in this meta-analysis involved patients in the stable maintenance period. One study showed that in patients on a combination of clozapine and olanzapine with worsening psychiatric symptoms, discontinuation of olanzapine did not worsen symptoms. Since the current study was regarding medication use during hospitalization, we assumed that most of the patients had significant psychotic symptoms. In this situation, antipsychotic dose reduction may be a treatment option worth considering, although special attention must be paid to the possibility of further exacerbation of the psychiatric symptoms.

The relationship between the main drug and the other aforementioned psychotropics was not observed for patients with MDD, while the number of antipsychotics, especially atypical antipsychotics, increased more often in the poly_mono group than in the poly_poly group. This result is consistent with the results of a recent study of 43,868 MDD inpatients, which showed that more patients were treated with combination therapy of one antidepressant plus atypical antipsychotics (23.8–74.8%, according to severity) than with polypharmacy of antidepressants (26.3–24.9%) [11]. Because the treatment guidelines recommended augmentation by mood stabilizers or atypical antipsychotics before prescribing polypharmacy of antidepressants [8, 9], switching from antidepressant polypharmacy to antidepressant monotherapy and augmentation with atypical antipsychotics may be appropriate.

No change in prescription between admission and discharge was observed in more than 10% of the patients with schizophrenia and MDD. Among patients with schizophrenia, those without any change in their prescription were significantly more likely to receive mECT. Among patients with MDD, mECT or CBT treatment did not differ significantly between those with a change in their prescription and those without prescription change. We do not have any other data on non-pharmacological therapy, and further studies are needed on the treatment of patients with MDD whose prescriptions did not change during admission.

Changing only supplemental psychotropics without any change in the main drug was more common among patients with MDD than among those with schizophrenia. This may reflect the treatment guideline recommendations. For schizophrenia, changing the main drug is the most important treatment strategy when the patient’s psychotic symptoms worsen [2, 3]. On the other hand, augmentation with mood stabilizers or atypical antipsychotics is recommended for the treatment of MDD [1, 9].

This study had several limitations. First, we did not assess the symptom severity of schizophrenia or MDD using rating scales such as the Brief Psychiatric Rating Scale, the Positive and Negative Syndrome Scale, Hamilton MDD Scale, or Montgomery-Åsberg MDD Rating Scale. Second, although we used a standardized data collection method and strict quality control, the data were collected from medical records obtained by the collaborating investigators in routine clinical settings, which might have affected the results. Third, although our data were gathered from all over Japan, and all participating sites were institutions that voluntarily participated in the study, and this could have resulted in the possibility of selection bias. There was also a bias in the types of hospitals, with nearly half of them being university hospitals, which might limit the generalizability of the results. To overcome this problem, we will continue our efforts to encourage more psychiatrists to take our courses and to collect prescribing data from a wider range of facilities in Japan. Forth, because we have not investigated the reasons for the change in prescription, we could not know whether the change in prescription was physician-initiated or at the request of the patient.

## Conclusions

In the current study, we assessed the changes in psychotropic prescriptions during hospitalization, before psychiatrists received special instructions regarding prescription guidelines. Our results showed that patients who received monotherapy with the main drug at admission were more likely to receive monotherapy at discharge and vice versa. In addition, when the main treatment involved a polypharmacy regimen, the number of psychotropic drugs prescribed besides the main treatment drug tended to increase. These results suggest that it is critical to avoid a polypharmacy regimen as the main treatment for MDD and schizophrenia. We gathered follow-up data from the same psychiatrists and assessed the changes in prescription patterns after these psychiatrists received special instructions regarding the guidelines. We expect clinicians to prescribe higher rates of monotherapy with the main drug after receiving special instructions regarding the guidelines.

## Electronic supplementary material

Below is the link to the electronic supplementary material.

Supplementary Material 1

## Data Availability

The datasets analyzed during the current study are not publicly available due to informed consent, which has not been obtained for the release of raw data, but are available from the corresponding author on reasonable request.

## List of Abbreviations

CBT: Cognitive behavioral therapy
EGUIDE: The Effectiveness of Guidelines for Dissemination and Education
MDD: Major depressive disorder
mono_mono group: Patients prescribed main drug monotherapy at admission and discharge
mono_poly group: Patients prescribed main drug monotherapy at admission and polypharmacy at discharge
poly_poly group: Patients prescribed polypharmacy of the main drugs at admission and discharge; and the
poly_mono group: Patients prescribed polypharmacy of the main drugs at admission and monotherapy at discharge
REAP-4AP: The Fourth Research on Asian Psychotropic Prescription Pattern
